# Cathepsin K regulates localization and secretion of Tartrate-Resistant Acid Phosphatase (TRAP) in TRAP-overexpressing MDA-MB-231 breast cancer cells

**DOI:** 10.1186/s12860-020-00253-6

**Published:** 2020-03-18

**Authors:** Anja Reithmeier, Maria Norgård, Barbro Ek-Rylander, Tuomas Näreoja, Göran Andersson

**Affiliations:** 1 Division of Pathology, Department of Laboratory Medicine, Karolinska Institutet, Alfred Nobels allé 8, 141 52 Stockholm, Sweden; 2 Present Address: Chemical Biology Consortium Sweden, Science for Life Laboratory Stockholm, Department of Medical Biochemistry & Biophysics, Karolinska Institutet, Tomtebodavägen 23A, 171 65 Solna, Sweden

**Keywords:** Cathepsin K, Tartrate-resistant acid phosphatase, *ACP5*, Proteolytic processing, Intracellular trafficking, Cancer

## Abstract

**Background:**

Tartrate–resistant acid phosphatase (TRAP/ ACP5) belongs to the binuclear metallophosphatase family and is present in two isoforms. The primary translation product is an uncleaved TRAP 5a isoform with low phosphatase activity. TRAP 5a can be post-translationally processed to a cleaved TRAP 5b isoform with high phosphatase activity by e.g. cysteine proteinases, such as Cathepsin K (CtsK). The relevance of the phosphatase activity of TRAP 5b has been demonstrated for proliferation, migration and invasion of cancer cells. TRAP-overexpressing MDA-MB-231 breast cancer cells displayed higher levels of TRAP 5a and efficient processing of TRAP 5a to TRAP 5b protein, but no changes in levels of CtsK when compared to mock-transfected cells. In TRAP-overexpressing cells colocalization of TRAP 5a and proCtsK was augmented, providing a plausible mechanism for generation of TRAP 5b. CtsK expression has been associated with cancer progression and has been pharmacologically targeted in several clinical studies.

**Results:**

In the current study, CtsK inhibition with MK-0822/Odanacatib did not abrogate the formation of TRAP 5b, but reversibly increased the intracellular levels of a N-terminal fragment of TRAP 5b and reduced secretion of TRAP 5a reversibly. However, MK-0822 treatment neither altered intracellular TRAP activity nor TRAP-dependent cell migration, suggesting involvement of additional proteases in proteolytic processing of TRAP 5a. Notwithstanding, CtsK was shown to be colocalized with TRAP and to be involved in the regulation of secretion of TRAP 5a in a breast cancer cell line, while it still was not essential for processing of TRAP 5a to TRAP 5b isoform.

**Conclusion:**

In cancer cells multiple proteases are involved in cleaving TRAP 5a to high-activity phosphatase TRAP 5b. However, CtsK-inhibiting treatment was able to reduce secretion TRAP 5a from TRAP-overexpressing cancer cells.

## Background

Tartrate-resistant acid phosphatase (TRAP/*ACP5*) is a metalloenzyme existing as two isoforms [1]. It is synthesized as the uncleaved highly glycosylated 35 kDa molecule TRAP 5a, containing a domain constituting a loop of around 20 amino acids, which is restricting its catalytic activity [2] and thereby retaining TRAP 5a in a latent low active state [3–5]. The isoform TRAP 5a can be cleaved by post-translational proteolytic excision to form isoform TRAP 5b with much higher phosphatase activity [3]. The cleaved protein consists of ~ 23 kDa/N-terminal and ~ 15 kDa/C-terminal subunit, connected via a disulfide bridge and missing the loop region present in TRAP 5a [3].

Unlike e.g. serine proteases that typically have high selectivity towards specific substrates [6] TRAP is relatively phosphosubstrate-unspecific. Therefore, its localization to vesicles and activation by proteolysis have been suggested as means to limit its function to a more specific set of substrates [7–10]. Several proteinases, such as trypsin and cysteine proteinases have been assessed for their potential to cleave and activate TRAP 5a [4, 11]. Cathepsin K (CtsK), an enzyme belonging to the class of cysteine proteinases, was shown to be very efficient in activating TRAP 5a enzyme by excision of the inhibitory loop domain in vitro [11], and producing a maximally active phosphatase similar to the cleaved bone-derived TRAP 5b [12]. CtsK was also colocalized with TRAP in osteoclasts [11]. Studies of osteoclasts of CtsK-deficient mice showed a major contribution of CtsK in proteolytic processing and intracellular trafficking of TRAP [13]. Furthermore, TRAP 5a has been suggested to function as a growth factor for cells of the mesenchymal lineage [14].

CtsK is a lysosomal enzyme identified to have high sequence homology to members of the papain-like cysteine protease superfamily including cathepsins S, L, and B [15, 16]. CtsK is produced as a catalytically inactive pre-pro-enzyme, which is activated upon autocatalyzed conversion to a mature enzyme. CtsK’s function was initially restrained to its expression in osteoclasts, where it degrades extracellular bone matrix proteins, such as fibrillar type-I collagen, and its ablation reduces bone resorption levels [15–17]. Studies during recent years have provided evidence that CtsK-levels are perturbed in a variety of pathological conditions. In particular, lack of CtsK function was associated to bone-related conditions such as osteopetrosis and the autosomal recessive gene disorder, pycnodysostosis [18], whereas overexpression of CtsK was reported in metastatic cancer cells [19–23]. A small chemical inhibitor of CtsK-activity, odanacatib/MK-0822 has been developed and it has shown to be an effective drug for osteoporosis and bone metastasis treatment within several clinical studies, however, some serious adverse effects have raised concerns. In summary, inhibition of CtsK reduced bone fracture rates and osteolysis, but also skeletal tumor burden, tumor volume and breast cancer invasion [18, 24, 25].

TRAP overexpression have been reported in several types of cancers [26–29]. Serum levels of TRAP isoform 5b are increased in several types of cancers with bone metastases [30–33], but the prognostic value of TRAP expression has been shown also in primary tumors [34]. Furthermore, inhibition of TRAP-activity by small molecule inhibitors normalized TRAP-induced migration caused by TRAP-overexpression in MDA-MB-231 cancer cells [35, 36]. Moreover, inhibition of CtsK in bone metastasis of breast cancer has been shown to reduce bone resorption [37]. Based on previous studies, we hypothesize that the highly active TRAP 5b isoform is correlated to aggressiveness in cancers, and that CtsK is involved in activation of TRAP by cleaving TRAP 5a to TRAP 5b. We present the effects of CtsK-activity on TRAP expression and function in a TRAP-overexpressing MDA-MB-231 cancer cell line. MK-0822 treatment affected cleaving patterns and secretion of TRAP-isoforms, but had no effect on activity of TRAP, TRAP isoform distribution and migration of the cancer cells.

## Results

### TRAP–overexpressing breast cancer cells generate elevated amounts of TRAP 5b

**Fig. 1:**
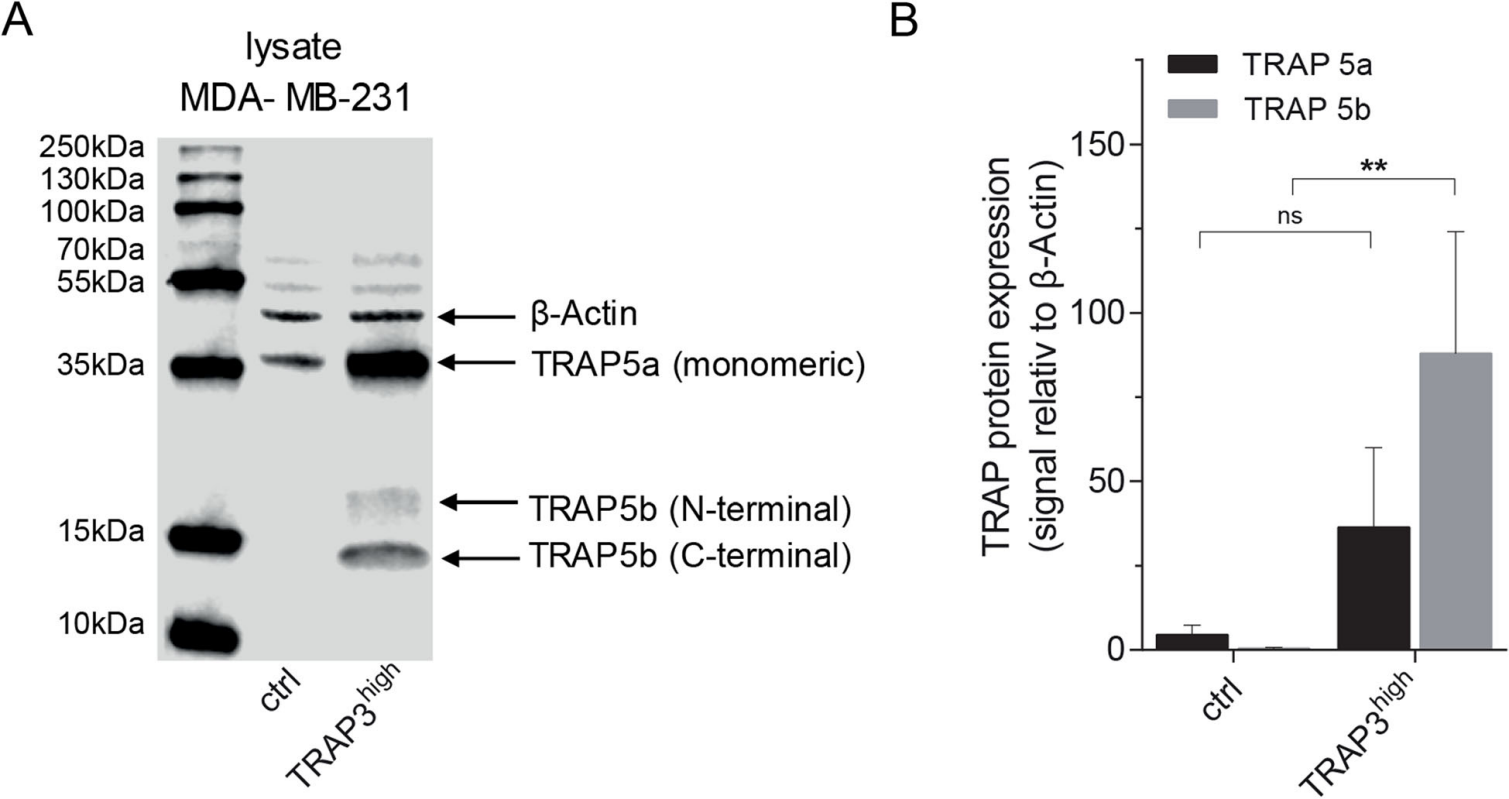
TRAP isoform prevalence in MDA-MB-231 cells. **a** One representative Western blot of TRAP isoform expression and processing is shown in mock control and TRAP3^high^ cell lysate. Membranes were stained with anti-total TRAP antibody and anti-β-Actin antibody. **b** Quantification of TRAP isoform 5a (uncleaved TRAP, 35 kDa) and 5b (cleaved TRAP, ~ 23 kDa/N-terminal and ~ 15 kDa/C-terminal) expression in mock control and TRAP3^high^ cell lysates from several Western blots with normalization to β-Actin (42 kDa) and statistically compared by ANOVA test (Fisher’s LSD test, independent experiments mock ctrl: *n* = 10, TRAP3^high^*n* = 9, bar graphs represent means and Standard error, B). ** *p* < 0.01

Our aim was to examine are the functional effects of overexpressed TRAP mediated by its high phosphatase-activity isoform TRAP 5b. Furthermore, it was investigated if inhibition of CtsK would inhibit proteolytic processing of TRAP, and also, would this be sufficient to abrogate the functional effects of TRAP-overexpression, such as increased migration. In a TRAP-overexpressing MDA-MB-231 breast cancer cells (TRAP3^high^) and mock-transfected control cells (mock ctrl) the total TRAP-isoform distribution was detected by Western blot. A polyclonal antiserum containing antibodies recognizing both the constitutive human TRAP and overexpressed rat TRAP 5a and TRAP 5b isoforms was used for the detection. Both mock and TRAP3^high^ cell clones expressed TRAP 5a, whereas TRAP 5b was only detectable in the TRAP3^high^ cells, as previously reported [35, 36] (Fig. 1a). By densitometric quantification of the signals, the TRAP-overexpressing MDA-MB-231 cells displayed higher TRAP 5a signal, however, due to the variation of expression over the culture period the difference was not significant between the lines (Fig. 1b). The low levels TRAP 5a detected in the mock control MDA-MB-231 cells came from endogenously expressed human TRAP, whereas TRAP 5a in the TRAP3^high^ cells derive both from the constitutive human TRAP 5a expression and the overexpressed rat TRAP. More importantly, in TRAP3^high^ cell lysates TRAP 5b amounts were significantly increased, while the TRAP 5b isoform was absent in mock control cells (Fig. 1b), suggesting that the bulk of the overexpressed rat TRAP 5a is efficiently converted to the cleaved 5b isoform.

### Expression of CtsK is not affected by TRAP-overexpression

**Fig. 2:**
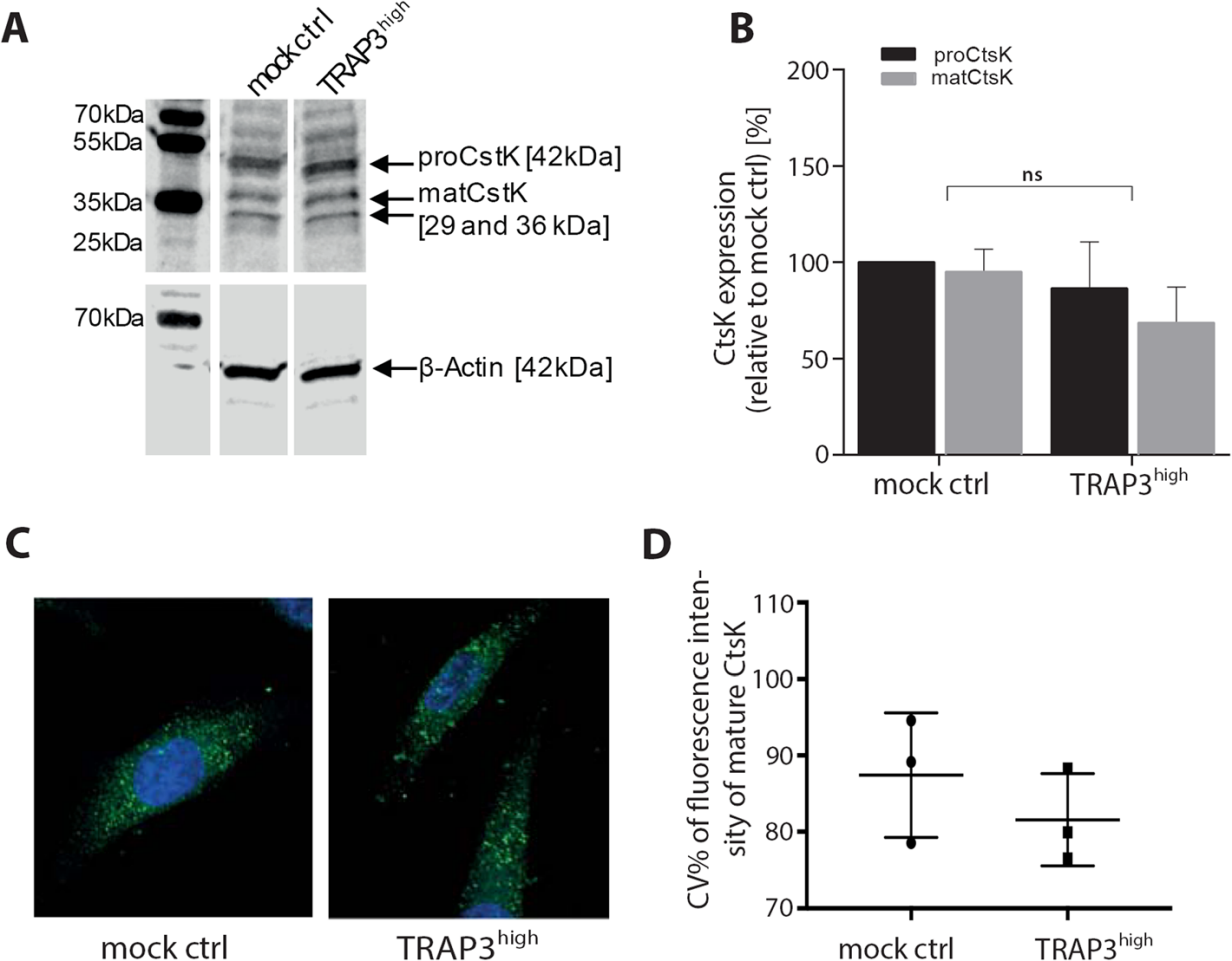
Cathepsin (CtsK) expression and distribution in MDA-MB-231 cells. **a** One representative Western blot of CtsK expression is shown from mock control and TRAP3^high^ cell lysate. Membranes were stained with anti-CtsK antibody and anti-β-Actin antibody. **b** Quantification of pro CtsK and mature CtsK (matCtsK) expression in mock control and TRAP3^high^ cell lysates from several Western blots with normalization to β-Actin (independent experiments *n* = 5, bar graphs represent means and Standard error) and statistically compared by ANOVA test (Fisher’s LSD test). **c** Immunocytochemistry (ICC) staining of total CtsK mock control and TRAP3^high^ cells (*n* = 3 independent cultures). Cells were stained with CtsK reactive rabbit antiserum (Alexa 488, green) and Hoechst (blue) and a representative maximum intensity projection is shown. **d** Coefficient of variation of Alexa 488 signal was calculated to analyze CtsK signal distribution over the cell. Means (8–20 cells in at least 3 fields of view) from 3 independent experiments were statistically compared by t-test

As the TRAP-overexpressing cells displayed an abundance of the proteolytically processed TRAP 5b isoform, we wanted to examine the role of the most likely cleaving protease, CtsK. CtsK was earlier shown to be involved in the processing of TRAP secreted by osteoclasts and pan-cysteine proteinase inhibition by E-64 was able to partly impair processing of TRAP 5a to TRAP 5b in MDA-MB 231 cells [38]. Furthermore, CtsK expression and activity have been reported in a variety of cancers and cancer cell lines leading to similar phenotype than high TRAP expression [20, 21, 23]. The presence of a positive feedback connection in expression of TRAP and CtsK was assessed, and therefore, CtsK expression levels were measured in the TRAP-overexpressing breast cancer cell line MDA-MB-231 and the corresponding mock control cells. Both mock control and TRAP3^high^ cells expressed easily detected amounts of proCtsK and mature CtsK (Fig. 2a) at similar concentrations (Fig. 2b), hence, the increase of CtsK levels could not explain TRAP 5b generation in TRAP-overexpressing cells.

Immunocytochemistry (ICC) (Fig. 2c) was used to localize CtsK in relation to the TRAP-isoforms to study further the role of CtsK in the intracellular processing of TRAP and in increased generation of TRAP 5b in TRAP-overexpressing cells. The intracellular localization and distribution of mature CtsK appeared, as expected vesicular, but also, similar in both mock and TRAP3^high^ cells (Fig. 2c). To describe differences in distribution of the proteins i.e. diffuse cytoplasmic localization or compartmentalized localization, signal coefficient of variation (CV%) per cell was quantified. Here, the higher the signal CV% the more compartmentalized the signal was to specific locations in a cell e.g. vesicles, whereas the lower the CV% the more uniformly diffuse the signal was spread throughout a cell e.g. cytoplasm. In ICC experiments a compartmentalized localization of mature CtsK was observed in both mock and TRAP-overexpressing cells (Fig. 2d), but no differences in compartmentalization between the cell lines were observed. Hence, it would appear that active CtsK in MDA-MB-231 cells was stored in intracellular compartments, and both expression and localization were unaffected by TRAP-overexpression.

### ProCtsK is highly co-localized with TRAP in TRAP-overexpressing breast cancer cells

**Fig. 3:**
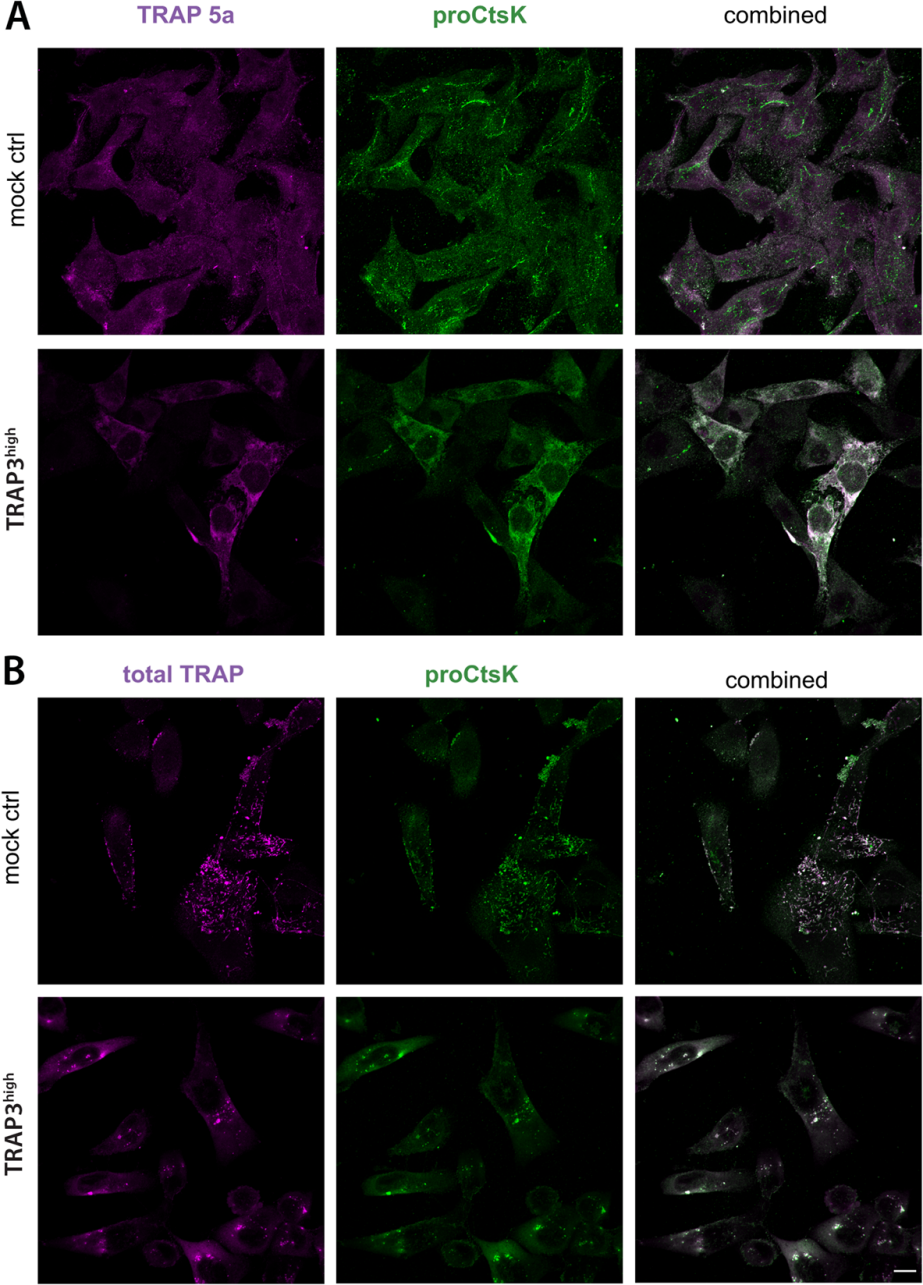
Co-staining of TRAP isoforms and proCtsK in MDA-MB-231 cells. **a** Immunocytochemistry (ICC) staining of proCtsK (Alexa 488, green) and uncleaved TRAP 5a (Alexa 647, magenta) of mock control and TRAP3^high^ cells (independent experiments *n* = 3). **b** ICC staining of proCtsK (Alexa 488, green) and total TRAP (Alexa 647, magenta) of mock control and TRAP3^high^ cells (independent experiments *n* = 3). Representative single color and merged maximum intensity projections are shown. Scale bar is 10 μm, all subpanels have the same magnification

**Fig. 4:**
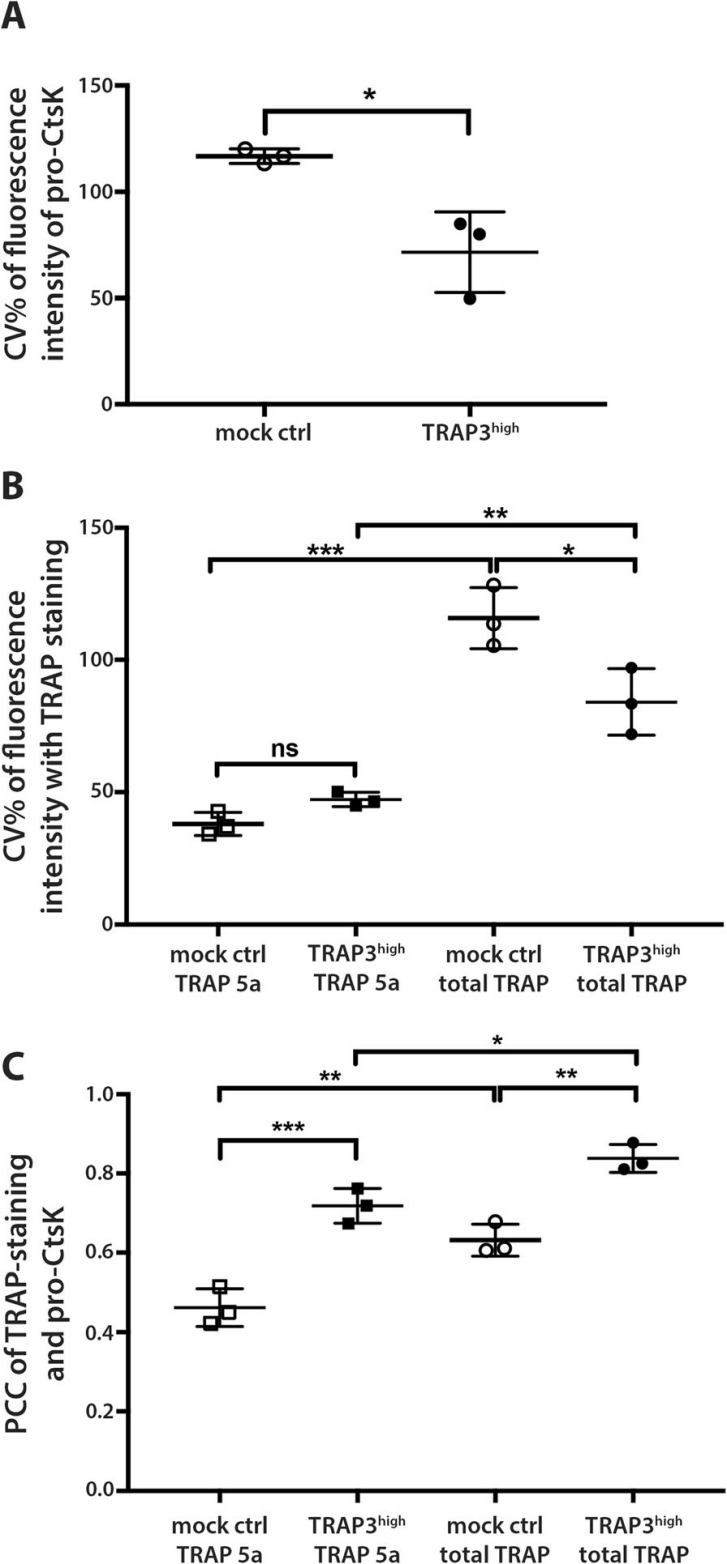
Distribution and colocalization of proCtsK and TRAP isoforms in MDA-MB-231cells. **a** Coefficient of variation of Alexa 488 signal was calculated to analyze distribution of proCtsK signal in the cell. Means (8–20 cells in at least 3 fields of view) from 3 independent experiments were statistically compared by t-test (*n* = 3 cultures). **b** Coefficient of variation of Alexa 647 signal (B) was calculated to analyze distribution of TRAP 5a and total TRAP signals over the cell. Means from independent experiments were statistically compared by one-way ANOVA. **c** Colocalization of proCtsK and uncleaved TRAP 5a vs. proCtsK and total TRAP was analyzed by calculation of a Pearson’s correlation coefficient (PCC). Means from independent experiments were statistically compared by one-way ANOVA with Tukey’s correction for multiple comparisons. * *p* < 0.05, ** *p* < 0.01 *** *p* < 0.001

However, there are several processing steps in activation of CtsK [23], and therefore, we co-stained the MDA-MB-231 cells also for proCtsK and TRAP-isoforms. Representative images of immunocytochemistry experiments stained with the aforementioned antibodies are depicted in Fig. 3 and Supplementary videos 1, 2, 3 and 4). Cells with high TRAP-signal in ICC, stained stronger also for proCtsK, and the cultures of TRAP-overexpressing cells displayed higher cell-to-cell variation (Fig. 3). Compartmentalization of proCtsK, expressed by a high CV%, was higher in mock control cells (Fig. 4a), while in cells with high amounts of TRAP a more diffuse localization of proCtsK was apparent (Fig. 3). This suggests that overexpression of TRAP in cancer cells was able to alter the distribution of proCtsK to facilitate generation of the TRAP 5b isoform.

To investigate the interdependence of TRAP and CtsK further, we examined the distribution of the uncleaved TRAP 5a and total TRAP (isoforms uncleaved 5a + cleaved 5b), and also, their relation to proCtsK. The staining for total TRAP (isoforms 5a + 5b) appeared to be more compartmentalized in both mock and TRAP3^high^ cells (Fig. 3b) than the staining for TRAP 5a alone (Fig. 3a). In TRAP3^high^ cells a number of vesicles stained with total TRAP antibody had a much higher fluorescence intensity compared with the other stained conditions. Concordantly, the total TRAP staining displayed significantly higher CV% in both mock control and TRAP3^high^ cells (Fig. 4b). The higher compartmentalization could be attributed to the presence of proteolytically cleaved TRAP 5b confined inside vesicles in TRAP3^high^ cells. However, only a slight increase in CV% of TRAP 5a was observed (Fig. 4b), hence, compartmentalization could serve as a prerequisite for further processing. Furthermore, compartmentalization did not always lead to further processing (Fig. 1)**,** as increased compartmentalization of total TRAP was observed also in mock control cells (Fig. 4b), suggesting that the apparent changes in distribution of the TRAP isoforms might be a result of another step in the processing of TRAP.

Therefore, the relation of these compartments to cleaving of TRAP 5a was investigated by co-localization measurements using Pearson’s correlation coefficients (PCC) between proCtsK and TRAP 5a as well as proCtsK and total TRAP (isoform 5a + 5b) (Figs. 3 and 4c). Consistent with the idea of CtsK being a TRAP-cleaving protease, a significantly higher degree of colocalization of both TRAP isoforms and proCtsK was observed in the TRAP3^high^ cells compared to the mock control cells (Fig. 4c). Colocalization of total TRAP with proCtsK was significantly higher in the TRAP3^high^ cells, indicated by the mean PCC values of 0.63 and 0.84 for mock control and TRAP3^high^ cells, respectively (Fig. 4c). Moreover, the PCC values of total TRAP and proCtsK were higher than those of TRAP 5a and proCtsK 0.46 in mock control and 0.72 in TRAP3^high^ cells (Fig. 4c). This is an indication of the cleaved TRAP 5b remaining in the proCtsK-positive compartments. Our data collectively suggests that more efficient TRAP 5a colocalization with proCtsK in TRAP3^high^ cells (Fig. 4 b) could explain the higher abundance of TRAP 5b generated in these cells (Fig. 1).

Nevertheless, while moderate level of colocalization was present also in the mock control cells, no TRAP 5b was produced (Fig. 1). Hence, the signal would need to originate from the same protein, and yet it exhibits a remarkably different localization pattern (Fig. 3). Furthermore, the higher colocalization of total TRAP and proCtsK compared to TRAP 5a and proCtsK, was apparent especially in the control cells, (Figs. 3 and 4c). Also, the compartmentalization of total TRAP measured as CV% in the mock control was significantly higher than in the TRAP-overexpressing cells (Fig. 4b).

### CtsK activity regulates localization and trafficking of TRAP-isoforms

**Fig. 5:**
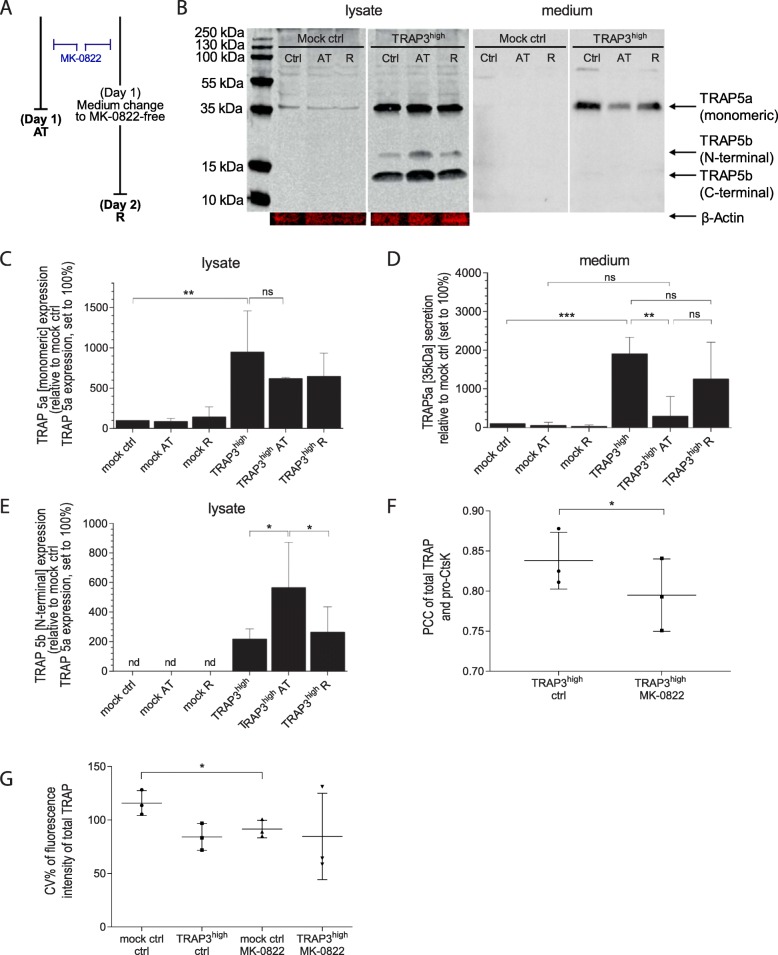
TRAP isoform prevalence after CtsK inhibition. **a** Cells were treated with 100 nM CtsK inhibitor (MK-0822, Odanacatib) and lysates and conditioned media were collected either directly after 24 h treatment (AT) or after an additional recovery period for 24 h after the treatment (R) at 48 h. Controls were treated with equal concentration of DMSO. **b** One representative Western blot of TRAP isoform expression and processing in mock control and TRAP3^high^ cells is shown. Membranes were stained with anti-total TRAP antibody and anti-β-Actin antibody in lysates. Quantification of TRAP isoforms in mock control and TRAP3^high^ cells **c** TRAP 5a 35 kDa in lysates, **d** TRAP 5a 35 kDa in conditioned media and TRAP 5b N-terminal subunits ~ 23 kDa) in mock control and TRAP3^high^ cell lysate. Western blots were normalized to β-Actin (independent experiments *n* = 3, bar graphs represent means and Standard error). Expression levels were normalized to TRAP 5a expression in the mock control cells and statistically compared by ANOVA test (Fisher’s LSD test). **f** Colocalization of total TRAP and proCtsK was measured in TRAP3^high^ cells in presence of MK-0822. Means (independent experiments *n* = 3, technical replicates 8–20 cells in at least 3 fields of view) from independent cultures were statistically compared by t- test. **g** Compartmentalization of total TRAP-signal, means from independent experiments of cells treated with MK-0822 were statistically compared by one-way ANOVA with Tukey’s correction for multiple comparisons. * *p* < 0.05, ** *p* < 0.01 *** *p* < 0.001

To understand the role of CtsK in TRAP processing, the cells were treated with a specific small molecule inhibitor of CtsK MK-0822 (Odanacatib). Changes in TRAP-isoform levels were measured in cell lysates after 24 h treatment (AT) (Fig. 5a). To study the reversibility of CtsK-inhibition, cell lysates and corresponding culture medium were extracted on day 2 after the medium was replaced with MK-0822 –free medium after 24 h to allow recovery (R). A representative Western blot is shown in Fig. 5b. Levels of TRAP 5a in lysates were higher in the TRAP3^high^ cells compared to the mock control cells, but were not significantly changed between the treatment conditions neither in mock control nor in TRAP3^high^ cells at AT or R (Fig. 5c). However, levels of TRAP 5a secreted to the medium were significantly lower under MK-0822 treatment (AT) and returned to normal levels after lifting the inhibition (R) (Fig. 5d). In the lysates, levels of TRAP 5b N-terminal subunit were significantly higher under inhibition (AT), and again, returned to control-levels after recovery from CtsK-inhibition (R) (Fig. 5e). Hence, CtsK is not necessary for cleaving TRAP 5a to TRAP 5b in breast cancer cells (Fig. 5e). More importantly, it appears to control the secretion of it, as less TRAP 5a is released to medium (Fig. 5d) under MK-0822 treatment. This suggests a regulatory role for CtsK in TRAP trafficking. Therefore, the effects of MK-0822 treatment were investigated by ICC-staining and measurement of colocalization and compartmentalization of total TRAP and proCtsK (Supplementary Figure 1). In the TRAP-overexpressing cells a decrease in colocalization of total TRAP and proCtsK was observed (Fig. 5f). This provides further evidence that colocalization with proCtsK is required before TRAP will be secreted, but this colocalization was not required for cleavage of TRAP. In the mock control cells, we observed that the compartmentalization of total TRAP was significantly decreased to levels similar to the TRAP3^high^ cells (Fig. 5g) and a smaller decrease was seen in the TRAP-overexpressing cells.

### CtsK is not essential in generating active TRAP 5b isoform

**Fig. 6:**
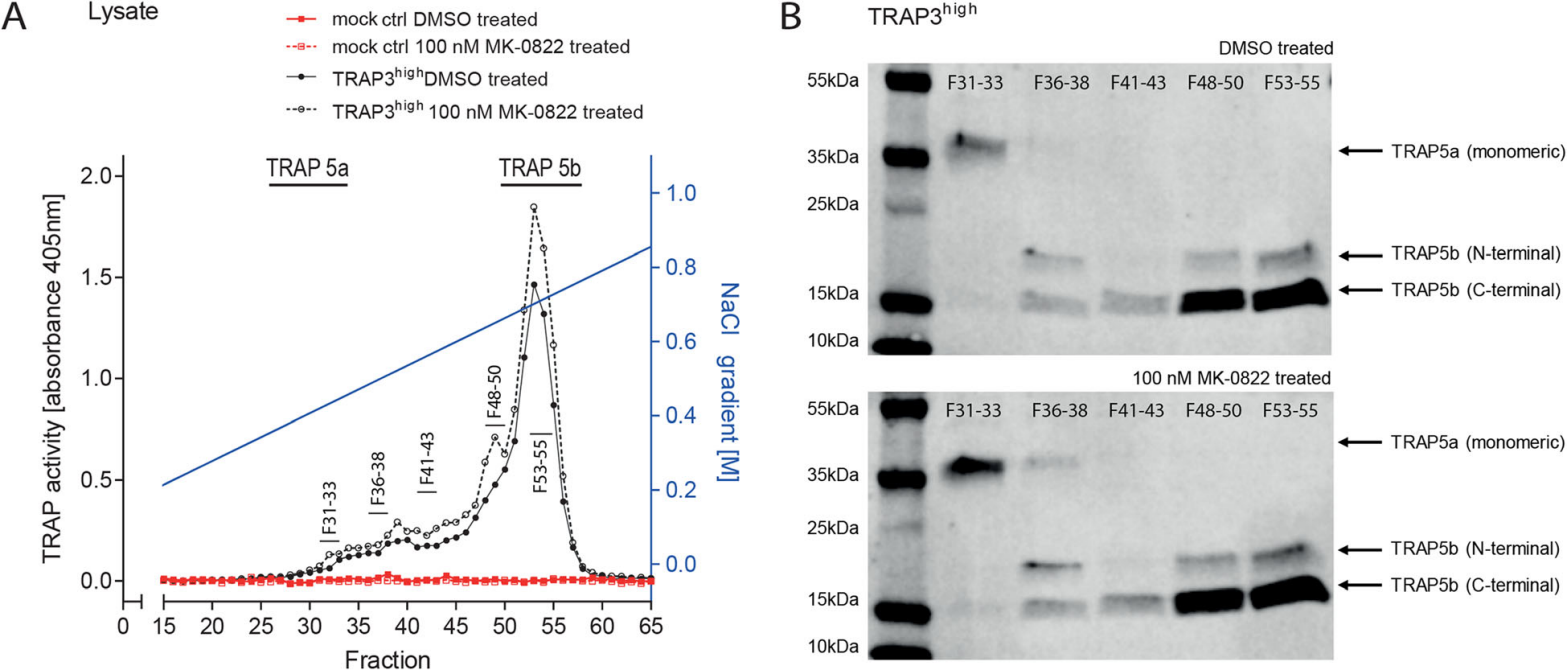
TRAP isoform fractioning in MDA-MB-231 cells after CtsK inhibition*.***a** Fast protein liquid chromatography (FPLC) of cell lysate derived from mock control and/or TRAP3^high^ cells treated with DMSO or with 100 nM CtsK inhibitor (MK-0822, Odanacatib) on heparin columns and a NaCl gradient from 0.1 M– 1 M NaCl (independent experiments *n* = 2). Fractions were measured for TRAP activity (represented by absorbance signal at 405 nm). **b** Respective peaks for FPLC pattern of recombinant TRAP 5a and TRAP 5b protein are denoted. Western blot for total TRAP protein expression. FPLC Fractions of TRAP3^high^ cells treated with DMSO or with 100 nM CtsK inhibitor (MK-0822, Odanacatib) were measured for TRAP protein expression and denoted as FXX-XX

To establish if activity of the cleaved TRAP 5b was altered after CtsK-independent cleavage we separated the TRAP isoforms by Fast Protein Liquid Chromatography (FPLC) and compared them to the fractions of recombinant TRAP 5a and TRAP 5b (not shown). DMSO control and MK-0822 –treated lysates were fractioned on heparin columns with a NaCl gradient and subsequently phosphatase activities of the fractions were measured (Fig. 6a). Low phosphatase activity was measured in all of the lysate fractions from the mock control cells. Lysates of the TRAP3^high^ cells, however, displayed TRAP activity from fraction 30, overlapping with the elution profile of the less active recombinant TRAP 5a isoform, and a high activity in fractions 50–56, overlapping with the elution profile of recombinant fully cleaved TRAP 5b. A similar pattern compared to the DMSO treated cells was detected in the lysates from TRAP3^high^ cells treated with the MK-0822. Distinct peaks with increased activity compared to the DMSO treated TRAP3^high^ cells were detected in earlier lysate fractions at 39 and at 49. In good agreement with higher TRAP 5b levels in MK-0822 treated cells, also the total TRAP activity was higher in lysate fractions 52–55 of MK-0822 treated cells compared with DMSO treated cells.

When selected fractions were pooled, concentrated and subjected to Western blotting (Fig. 6b), fractions 31–33 contained mainly TRAP 5a, whereas cleaved TRAP 5b was detected from fractions 36–55, with increasing protein amount from fraction 48 in both control and MK-0822 treated lysates. Minor amounts of TRAP 5a were still detectable in fractions 36–38 in the MK-0822 treated cells, whereas absent in DMSO treated cells and higher band intensities of TRAP 5a were detected in the MK-0822 treated cells compared to the DMSO control cells in F31–33 and F36–38 (Supplementary Table 1). Interestingly, several band sizes were present at the TRAP 5b isoforms and fractions 36–38 and 48–50 displayed increased protein intensities at the N-terminal TRAP 5b band in the MK-0822 treated cell fractions compared to the DMSO treated cells (Supplementary Table 1). Put together, it appears that under CtsK inhibition the cells still accumulate the cleaved TRAP 5b isoforms (Fig. 5e), but the TRAP 5b might not be fully processed i.e. the loop can be cleaved only from one side and still remains on the cleaved TRAP 5b.

### CtsK inhibition does not reduce TRAP activity or TRAP-dependent migration

**Fig. 7:**
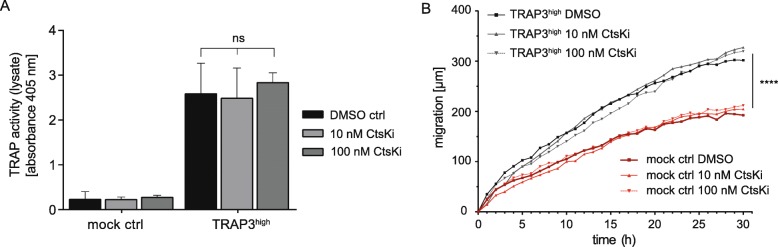
Cellular TRAP activity and wound migration after CtsK inhibition by MK0822. **a** Cells were treated with 10 nM and 100 nM CtsK inhibitor (MK-0822, Odanacatib), and lysates collected after 24 h of treatment. Total TRAP activity (represented by absorbance signal) from cell lysate derived from mock control or TRAP3^high^ cells. Averages from *n* = 3 independent experiments were statistically compared by ANOVA test (Fisher’s LSD test). **b** Live cell wound migration of TRAP-overexpressing (TRAP3^high^, black) and control (ctrl, red) MDA-MB-231 cells under treatment with the CtsK inhibitor (10 nM and 100 nM of MK-0822, Odanacatib, independent experiments *n* = 3, 4 or more technical replicates each). Statistical comparison in GraphPad Prism 6 software was performed by ANOVA (Fisher’s LSD test) on migration velocity assessed by linear regression analysis between normally distributed groups. * *p* < 0.05, *** *p* < 0.001

As N-terminal TRAP 5b protein levels were increased when CtsK was inhibited, concordantly with an increase in late TRAP 5a fractions, we were interested to find out if inhibition of CtsK would alter total cellular TRAP activity. Inhibition of CtsK with MK-0822 did not modulate TRAP activity in lysates at concentrations of 10 nM and 100 nM (Fig. 7a). Concordantly with the fact that TRAP activity was unchanged, inhibition of CtsK did not modulate TRAP-dependent migration (Fig. 7b). This indicates that even under MK-0822-treatment the cleaved TRAP 5b is biologically active and exerts its intracellular function on cell migration.

## Discussion

Earlier studies have investigated several cancer cell types for TRAP expression and shown an association between TRAP expression and cancer aggressiveness [26, 27, 39, 40]. Also, TRAP expression has been shown to correlate with poor prognosis in clinical studies [28–34]. Moreover, blocking of TRAP activity by targeting the TRAP 5b isoform with a small molecule inhibitor abrogated metastasis-related cancer cell functions in TRAP-overexpressing MDA-MB-231 breast cancer cells [35]. Here, we investigated CtsK as a potential activator of TRAP processing, localization and trafficking, as well as functional activity in a TRAP-overexpressing cancer cell line that has been previously characterized [36]. TRAP 5b levels were significantly increased in the TRAP-overexpressing cells compared to the mock control cells when respective amounts of the TRAP-isoforms were measured. The TRAP 5b isoform has higher phosphatase activity when compared to the TRAP 5a isoform [11], thus higher amounts of TRAP 5b most likely account for the higher total cellular TRAP activity in these cells, as shown by the FPLC analysis.

TRAP 5b is generated from TRAP 5a by an activating proteolytic cleavage, as observed in osteoclasts, a cell type expressing high levels of TRAP [1]. In osteoclasts, CtsK was the principal enzyme mediating proteolytic cleavage of TRAP 5a to TRAP 5b and was highly expressed and colocalized with TRAP [11, 15, 22, 41, 42]. Furthermore, CtsK^−/−^ knockout mice displayed increased ratios of TRAP 5a to TRAP 5b in bone lysates [13], and both CtsK and CtsL can cleave and activate TRAP in vitro [11]. This led to the hypothesis that CtsK also mediates TRAP processing, i.e. generation of high TRAP 5b levels in the MDA-MB-231 TRAP-overexpressing cancer cells. Specific inhibition of CtsK in osteoclasts [43] has been shown to increase the intracellular TRAP amounts and inhibition of all cysteine proteases in MDA-MB-231 [38] cells, and osteoclasts [23] has been shown to modulate proportions of TRAP 5a and TRAP 5b. However, nether treatment was fully able to inhibit cleavage of TRAP. However, functional effects of reduced CtsK function are observed in osteoclasts [43, 44], suggesting that osteoclasts may be more sensitive to differences in TRAP-isoforms compared to other cell types.

High expression of cysteine proteinases and in particular of CtsK has been detected in a variety of cancer cells. Amongst several other cancer cell lines, CtsK expression was detected also in the MDA-MB-231 breast cancer cells [20–23]. As neither pro- nor mature CtsK expression was different between the mock control and the TRAP-overexpressing cells on whole cell lysate levels, an increased TRAP-processing due to TRAP-dependent increase of CtsK levels induced by higher TRAP-levels could be excluded. However, in the ICC-experiments individual cells with both high proCtsK and TRAP 5a were observed indicating that there could be some fluctuations over time in the expression of both proteins. Nevertheless, the ICC-signals from staining detecting both pro- and matCtsK were similarly restricted to sub-cellular compartments in the mock control and the TRAP-overexpressing cells, whereas signal from the staining detecting only proCtsK was significantly more compartmentalized in the mock control cells than in the TRAP3^high^ cells. Interestingly, both proCtsK and mature CtsK were tightly compartmentalized, while compartmentalization of uncleaved TRAP 5a was low in both mock control and TRAP3^high^ cells. Furthermore, colocalization of proCtsK and TRAP 5a was higher in TRAP3^high^ cells than in mock control cells that supports a CtsK’s role in cleaving TRAP 5a to TRAP 5b. Moreover, both higher compartmentalization of total TRAP (isoforms 5a + 5b) staining and tighter colocalization of proCtsK and total TRAP compared with similar experiments with TRAP 5a indicate a close association of the two proteins in membrane enclosed sites where active proteolytic post-translational processing takes place, e.g. lysosomes.

However, involvement of CtsK in the cleavage of TRAP, is not able to explain the observations in the mock control cells: namely that tighter colocalization of total TRAP and proCtsK compared to colocalization of TRAP 5a and proCtsK and that the TRAP 5b is completely absent in the lysate and the discrepancy between staining for TRAP 5a and total TRAP. In the mock control cells antibody for TRAP 5a did not stain the proCtsK structures whereas the total TRAP antibody did, indicating that the interaction of the two proteins could make the loop-domain inaccessible to the antibody or another structural feature prevents the interaction. A study of TRAP 5a crystal structure has revealed that the inhibitory loop-domain can exist in either an open or closed conformation [45]. The discrepancy between the ICC signals could thus indicate that the TRAP-loop antibody was unable to recognize a closed conformation of the loop-domain and that in the mock control cells, a large proportion of the compartmentalized TRAP 5a was in the closed conformation, and thus possibly also less accessible to proteolytic processing. The pattern in the TRAP3^high^ cells is more ambiguous as they contain also TRAP 5b where the loop-domain has been cleaved, but also in these cells the signal from TRAP-loop antibody was more diffusely distributed. Therefore, an open conformation of the loop-domain might serve as a signal to compartmentalize TRAP. As proCtsK has always higher compartmentalization, the interaction of TRAP 5a and proCtsK appears to incur after TRAP 5a has been compartmentalized.

In CtsK knockout mice, the secretory pathway of TRAP in osteoclasts was disturbed [13]. Furthermore, small molecule inhibition of CtsK was reported to disrupt vesicular trafficking in osteoclasts [43]. When the MDA-MB-231 cells were treated with a CtsK-specific inhibitor MK-0822 (Odanacatib), a slight decrease in colocalization was observed in the TRAP3^high^ cells and a decrease in compartmentalization of total TRAP signal in the mock control cells. This indicates that targeting of proCtsK and TRAP to the compartments where they undergo proteolytic post-translational processing occurs independent of CtsK-activity. The most striking effects, however, were observed in the trafficking of TRAP 5a. The intracellular TRAP 5a levels did not increase as a result of CtsK-inhibition; instead the N-terminal subunit of TRAP 5b accumulated inside the TRAP3^high^ cells. This indicates that CtsK is not necessary for cleaving the loop-region on TRAP 5a in the MDA-MB-231 cells, unlike in osteoclasts [11]. Concomitant with accumulation of TRAP 5b a decrease in secreted TRAP 5a was observed. Decreased secretion of TRAP 5a could provide more 5a isoform for intracellular proteolytic cleavage, thus potentially explaining the increased 5b levels in MK-0822–treated cells. These data together suggest that CtsK-inhibition indirectly could affect vesicular trafficking, possibly by altering processing of TRAP. This is in line with earlier publications where TRAP has been suggested to be a regulator of lysosomal protein transport [46] and where TRAP-deficient mice displayed a disturbed vesicular transport in osteoclasts [47].

A shift in elution of high phosphatase activity FPLC fractions including TRAP 5b closer to the fractions including low activity TRAP 5a was observed in the cancer cells. Similar results were reported earlier in the TRAP-overexpressing MDA-MB-231 cells, when treated with the pan-cysteine proteinase inhibitor E64 [38]. Also here, blocking with E64 was not able to totally abrogate the processing of TRAP 5b, but shifted TRAP 5b FPLC peaks to earlier eluting fractions [38]. Furthermore, CtsK-inhibition with MK-0822 did not decrease the amount of fully cleaved TRAP 5b in the FPLC fractions, in accordance with data from experiments with a Cathepsin B/L inhibitor [38]. However, the pan-cysteine proteinase inhibitor E64 was able to decrease also levels of TRAP 5b protein [38], suggesting that additional cysteine proteinases besides cathepsin B/L and CtsK are involved in cleaving TRAP 5a. The relative increase in the N-terminal band in fractions 36–50 most probably reflects partially cleaved or intermediate forms of the TRAP 5b isoform that might retain the loop region loosely associated with the N-terminal cleavage fragment and thus elute at earlier FPLC fractions, more similar to the elution profile of TRAP 5a. Nevertheless, increase of the N-terminal TRAP subunit levels did not change total physiological activity, as CtsK-inhibition was not sufficient to inhibit TRAP-dependent migration, consistent with previous reports [38]. Moreover, TRAP 5b appears to be exclusively responsible for driving TRAP-dependent migration.

## Conclusion

Put together, this evidence would suggest that both TRAP and CtsK are packed in the same compartments, and that these compartments are hubs of proteolytic processing of TRAP and other proteins. The compartments are poised to release their cargo to the plasma membrane or to other compartments with proteolytic function, and this trafficking is partly regulated by CtsK-activity.

This study showed that CtsK is involved in processing and trafficking of TRAP-isoforms in the TRAP-overexpressing MDA-MB-231 breast cancer cells. Also, association of proCtsK and TRAP in the TRAP3^high^ cells appears to correlate with compartmentalization of TRAP. Active CtsK is needed for secretion of TRAP 5a, suggesting a regulatory role for CtsK in secretion of proteins in vesicular compartments containing TRAP 5a. However, CtsK was not necessary for production of active TRAP 5b and inhibition of CtsK did not affect TRAP-dependent promotion of migration. Therefore**,** CtsK-inhibiting treatments of cancer progression are not likely to be mediated through TRAP-processing within cancer cells, but through decrease in secretion of growth factor–like TRAP 5a.

## Methods

### Materials

#### Primary antibodies

Rabbit anti-total TRAP (isoform 5a + 5b): polyclonal antibody serum, derived from New Zealand rabbits immunized with purified recombinant uncleaved TRAP 5a [12] (Western blotting 1:1000; TRAP 5a: 35 kDa, TRAP 5b: ~ 15 kDa C- and ~ 23 kDa N-terminal // immunocytochemistry 1:400); rabbit anti-uncleaved TRAP 5a: polyclonal antibody serum, derived from New Zealand rabbits immunized with the Loop-peptide ((DDFASQQPKMPRDLG-VAC–KLH (carrier)) [12, 48] (immunocytochemistry 1:300); rabbit anti-Cathepsin K (CtsK): polyclonal antibody serum, derived from rabbits immunized with a peptide (CGITNMASFPKM) as previously described [49] (Western blotting 1:1000, proCtsK: 42 kDa; mature CtsK: 19 kDa and 36 kDa; immunocytochemistry 1:400); goat anti-proCathepsin K: polyclonal antibody (immunocytochemistry 1:400, Abcam, Cat# ab77396; C-KTHRKQYNNKVDE); mouse anti-β-Actin: monoclonal antibody (Western blotting 1:1000: 42 kDa; Abcam; Cat# 8224).

#### Secondary antibodies

Donkey anti-mouse (Western blotting, 1:15000 Licor IRDye® 800CW, Cat#925–32,212); donkey anti-rabbit (Western blotting, 1:15000; Licor IRDye® 680RD, Cat# 926–68,073), donkey anti-goat Alexa Fluor 488 (immunocytochemistry 1:300, 2 mg/ml, Invitrogen); goat anti-rabbit Alexa Fluor 647 and 488 (immunocytochemistry 1:300, 1 mg/ml, Pierce, Thermo Scientific).

#### Inhibitory compounds

CtsK inhibitor Odanacatib (10 nM and 100 nM; MK0822; Catalog number S1115, Selleckchem; CAS No. 603139–19-1).

#### Recombinant enzyme

Human TRAP 5a purified from a concentrated baculovirus-infected *Spodoptera frugiperda* (Sf9) insect cell culture supernatant within a ÄKTA purifier™ 10 Fast protein liquid chromatography system with a protocol based on several sources [12, 38, 50] and as previously described [35]. TRAP was proteolytically cleaved as previously described [51]. Briefly, 0.1 μg/μL of human (Sf-9) recombinant TRAP 5a was incubated with 1.5 μg/μL of human cathepsin L (122,000 U/μL Calbiochem) for 3 h at 37 °C in 2 mM DTT, 20 mM NaOAc buffer (pH 5.5) and 1 mM EDTA. Reaction was terminated by adding 10 μg/ml E-64 (Boeringer-Mannheim) and aliquots frozen at − 20 °C.

### Cell line and culture

MDA-MB-231 breast cancer cells, derived from the American Type Culture Collection (Manassas, U.S., ATCC® Number: HTB-26™) have been stably transfected with the full rat TRAP insert [38] and subclones generated by single cell cloning. Rat TRAP was selected for its high (94%) amino acid sequence similarity to human TRAP while it still allowed for specific targeting by siRNA. In the loop region there was only amino acid type altering change between human and rat forms (R174M). Subclones have been characterized for TRAP expression and enzyme activity and the subclone TRAP3^high^ used for further studies, as it expressed high amounts of TRAP [36]. Cells were cultured in complete medium (RPMI 1640, 10% fetal bovine serum, 0.1 mg/mL Gentamicin) (Life technologies, Carlsbad, CA, U.S.) at 37 °C in a 5% CO_2_ humidified atmosphere. The cells were continuously tested for contamination with the MycoAlert™ mycoplasma detection kit (Lonza, Cat# LT07).

### Cell lysates

Protein lysates were prepared from 2-5 × 10^6^ cells grown in complete medium (RPMI 1640 supplemented with 0.1 mg/mL Gentamicin and 10% fetal bovine serum) (Life technologies). Before treatment, the cells were allowed to attach and expand for at least 24 h. After that, the medium was replaced with fresh serum-supplemented medium, respectively containing the small chemical CtsK inhibitor (MK-0822/Odanacatib) or DMSO (Sigma) as control. Lysates were prepared either after 24 h treatment (AT) or after an additional recovery time of 24 h without the inhibitor (R). For Western blotting cell pellets were lysed in 100 μL cold RIPA-buffer (100 mM Tris-HCl pH 8, 300 mM NaCl, 2% NP-40, 2% SDS, 1% Sodium Dodecyl Sulphate) per 10^6^ cells. For enzyme activity assays and Fast protein liquid chromatography (FPLC) analysis, lysates were prepared in homogenization buffer (0.15 M KCl, 0.1% Triton X-100) and 100 μL lysis buffer applied per 10^6^ cells. Here, lysates were collected only after 24 h treatment (AT).

All lysates were freshly supplemented with complete protease inhibitor cocktail (Roche Diagnostics, Basel, Switzerland) and homogenized by processing through a syringe. Protein debris was removed by centrifugation and total protein content was determined by the use of the Micro BCA Protein Assay Kit (Thermo Scientific) according to manufacturer’s descriptions.

### Western blotting

50 μg of total protein in lysates or similar volumes of Fast protein liquid chromatography (FPLC) purified and concentrated TRAP samples were applied to SDS-PAGE (Mini-PROTEAN® TGX™ precast gel, Biorad) and protein bands separated. Proteins were transferred to PVDF membranes (Trans-Blot turbo mini PVDF packs, Biorad) according to the manufacturer’s instructions. Unspecific binding was blocked by 3% bovine serum albumin (BSA) in PBS at room temperature (R.T.) for 1 h and protein bands stained by subsequent incubation with respective primary antibodies in 3% BSA in PBS overnight (o.n.) at 4 °C. After three washes in TBST (20 mM Tris-HCl pH 7.5, 500 mM NaCl, 0.05% Tween-20) for each 10 min, the membranes were incubated with fluorescently labelled IRDye® secondary antibodies for 1 h at R.T. The membranes were again washed for 3 times 10 min with TBST and subsequently visualized in the Licor Odyssey Fc Imager. Images were analyzed using the Licor Image Studio software 3.1.4 (Licor Biosciences, Lincoln, NE, U.S.) and protein quantities measured by fluorescent intensities in relation to respective amounts of β-Actin expression (in lysates). In media comparison was made on background subtracted fluorescence intensities.

### Immunocytochemistry, colocalization and cellular distribution analysis

Thirty-to fifty thousand cells were grown in coverslip thick glass chambers (IBIDI 8-well) in serum-supplemented medium for 24 h. The cells were then washed with PBS for 10 min and fixed with 4% paraformaldehyde. After permeabilization with 0.1% Triton X-100 in PBS for 15 min at R.T., unspecific binding was blocked by shaking in 0.1% BSA in PBS for another 60 min at R.T. The cells were then stained o.n. at 4 °C for TRAP isoforms and CtsK or proCtsK using the respective antibodies. Wells were washed in PBS for 3 × 10 min and the cells stained with respective secondary antibodies along with Hoechst 33342 (1:7000) o.n. at 4 °C. Unbound antibodies were washed away for 3 × 10 min in PBS and the slides mounted with Dako mounting medium. Z-stacks were captured with a NikonA1+ confocal laser microscope system using 100X objective fulfilling Nyquist sampling theorem. Laser power and detector gain were adjusted to cover widest possible range of intensity values for calculation of Pearson’s correlation coefficient (PCC). Nonspecific binding of secondary antibodies was low and imaging parameters were adjusted to leave nonspecific binding and autofluorescence of the samples below detection limit (Supplementary figure 2). 3D colocalization was measured per cell from the entire captured z-stacks as PCC with the NIS elements 4.3 colocalization macro. The pictures shown are z-stacks processed to maximum intensity projections. Intracellular distribution was measured from a sum-projection of the z-stacks as coefficient of variation (CV%) (100 x standard deviation of signal intensity (i.e. distribution of gray values in a cell) / mean intensity) in the TRAP or CtsK signals obtained from a single cell ROIs encompassing the cytoplasm i.e. not counting the nucleus. For each condition 8–20 cells were measured in multiple images and at least *n* = 3 independent experiments i.e. cell cultures originated from separate batches of frozen cells. The higher the CV% is, the more vesicular the localization appears to be, and the lower the CV% the more uniformly TRAP is distributed in the cytoplasm.

### Fast protein liquid chromatography (FPLC)

Recombinant human TRAP 5a or TRAP isoforms derived from cell lysates were separated as described previously [38, 50]. Briefly, an ÄKTA-purifier™ 10 FPLC system (GE Healthcare, Sweden) was applied with a Heparin column (Pharmacia) at a flow rate of 2 mL/ min at 4 °C. The Heparin column was equilibrated with 20 mM Tris-HCl (pH 7.2), 0.1 M NaCl, 0.005% Triton X-100 (w/v) and the protein in lysis buffer or medium eluted with a linear gradient of NaCl from 0.1 M – 1 M in 20 mM Tris-HCl (pH 7.2), 0.005% Triton X-100 (w/v) and collected in fractions of 1 mL each. FPLC spectra were compiled by measuring TRAP enzymatic activity per fraction and fractions further pooled and concentrated with Amicon® Ultra 15 mL Centrifugal Filters (MerckMillipore) for protein expression studies.

### TRAP enzymatic assay

A pNPP assay has been performed based on a modulated protocol previously described in [36]. The assay has been adapted to 96-well format by increasing assay volumes. Briefly, cell lysates were prepared by using 100 μL of lysis buffer per 10^6^ cells. The enzymatic reaction was allowed in a KCl-buffer under reducing conditions (0.15 M KCl, 0.1 M sodium acetate pH 5.8, 0.1% (v/v) Triton X-100, 10 mM Sodium tartrate, 1 mM Sodium ascorbate, 0.1 mM Fe (NH_4_)_2_(SO_4_)_2_) and TRAP-specific activity measured upon distinction from other tartrate-resistant acid phosphatases by addition of 100 μM sodium molybdate. To start the enzymatic reaction, 75 μL of pNPP substrate solution was added to 25 μL of enzyme solution and incubated for 60 min at 37 °C. The enzymatic reaction was stopped by addition of 100 μL 0.6 N NaOH solution. Enzymatic activity was measured by quantification of the absorbance of the end product para-nitrophenol (pNP) at 405 nm after phosphate release by the enzyme.

### Live cell wound migration

Live cell imaging experiments have been performed as previously described [35]. Briefly, 50,000 cells were seeded in half-area 96-well plates with a glass bottom (Corning 4580 high content imaging plate). Cells were allowed to grow under normal cell culture conditions for 48 h, including medium refreshment after 24 h. For CtsK inhibition MK-0822 was added to the medium for 24 h before migration start. At start of migration, wounds were generated, the cells carefully washed with pre-warmed migration medium (RPMI 1640, 1 mg/ mL bovine serum albumin (BSA) and 0.1 mg/ mL gentamicin) and migration medium added, respectively containing MK-0822. Wound migration was followed for 30 h by collecting an image series of 121 times points at 4X magnification in a NikonA1+ confocal laser microscope system equipped with temperature- and CO_2_-regulation. Migration distances were calculated by image processing and calculations performed with use of the NIS-Elements Advanced imaging software 4.3.0 (Nikon, Tokyo, Japan). Migration velocity was assessed by linear regression analysis in GraphPad Prism 6.

### Statistical comparisons

Statistical comparisons were performed in the GraphPad Prism software version 6.0. Tests for significant differences between normally distributed groups were performed by ANOVA test (uncorrected Fisher’s LSD) in case, when Western blot data of more than two groups were compared. For comparisons of two groups, paired t-test was applied. For colocalization and compartmentalization studies means from independent experiments were statistically compared by one-way ANOVA with Tukey’s correction for multiple comparisons. In all data, a minimum of three independent experiments (shown data points) were conducted. In ICC, each independent experiment had three technical replicates, of which a mean value was calculated to represent the experiment. Error bars represent ± SEM.

## Supplementary information

**Additional file 1: Supplementary material.** Including co-staining of TRAP isoforms and proCtsK in MDA-MB-231 cells treated with MK-0822. Densitometric quantification of Western blot TRAP bands of the different FPLC fractioned cell lysates. Controls for nonspecific binding of secondary antibodies.

**Additional file 2: Supplementary video 1 mock ctrl TRAP 5a.** Z-sections through ICC-stained proCtsK (Alexa 488, green) and uncleaved TRAP 5a (Alexa 647, magenta) mock control cells displayed in Fig. 3 as a maximum intensity projection.

**Additional file 3: Supplementary video 2 TRAPhi TRAP 5a.** Z-sections through ICC-stained proCtsK (Alexa 488, green) and uncleaved TRAP 5a (Alexa 647, magenta) TRAP3^high^ cells displayed in Fig. 3 as a maximum intensity projection.

**Additional file 4: Supplementary video 3 mock ctrl totalTRAP.** Z-sections through ICC-stained, proCtsK (Alexa 488, green) and total TRAP (Alexa 647, magenta), mock control cells.

**Additional file 5: Supplementary video 4 TRAPhi totalTRAP.** Z-sections through ICC-stained, proCtsK (Alexa 488, green) and total TRAP (Alexa 647, magenta), TRAP3^high^ cells.

## Data Availability

All raw-data relevant for this manuscript will be made available upon request.

## Abbreviations

CtsK: Cathepsin K
CV%: Coefficient of variation
DMSO: Dimethyl sulfoxide
FPLC: Fast protein liquid chromatography
ICC: Immunocytochemistry
PCC: Pearson’s correlation coefficient
TRAP: Tartrate-resistant acid phosphatase
