# Supplementary material for: Cathepsin K regulates localization and secretion of Tartrate-Resistant Acid Phosphatase (TRAP) in TRAP-overexpressing MDA-MB-231 breast cancer cells
Source: BMC Mol Cell Biol. 2020 Mar 18;21:15. doi: 10.1186/s12860-020-00253-6 (PMC7081696; doi:10.1186/s12860-020-00253-6)
Supplement: Supplementary file 1 — Additional file 1: Supplementary material. Including co-staining of TRAP isoforms and proCtsK in MDA-MB-231 cells treated with MK-0822. Densitometric quantification of Western blot TRAP bands of the different FPLC fractioned cell lysates. Controls for nonspecific binding of secondary antibodies. [file 12860_2020_253_MOESM1_ESM.pdf]

Cathepsin K regulates localization and secretion of Tartrate-Resistant Acid Phosphatase (TRAP) in TRAP-overexpressing MDA-MB-231 breast cancer cells

Anja Reithmeier[#,*], Maria Norgård, Barbro Ek-Rylander, Tuomas Näreoja[#,£], Göran Andersson[£]

Department of Laboratory Medicine, Division of Pathology, Karolinska Institutet, Huddinge 141 52, Sweden

Supplementary material

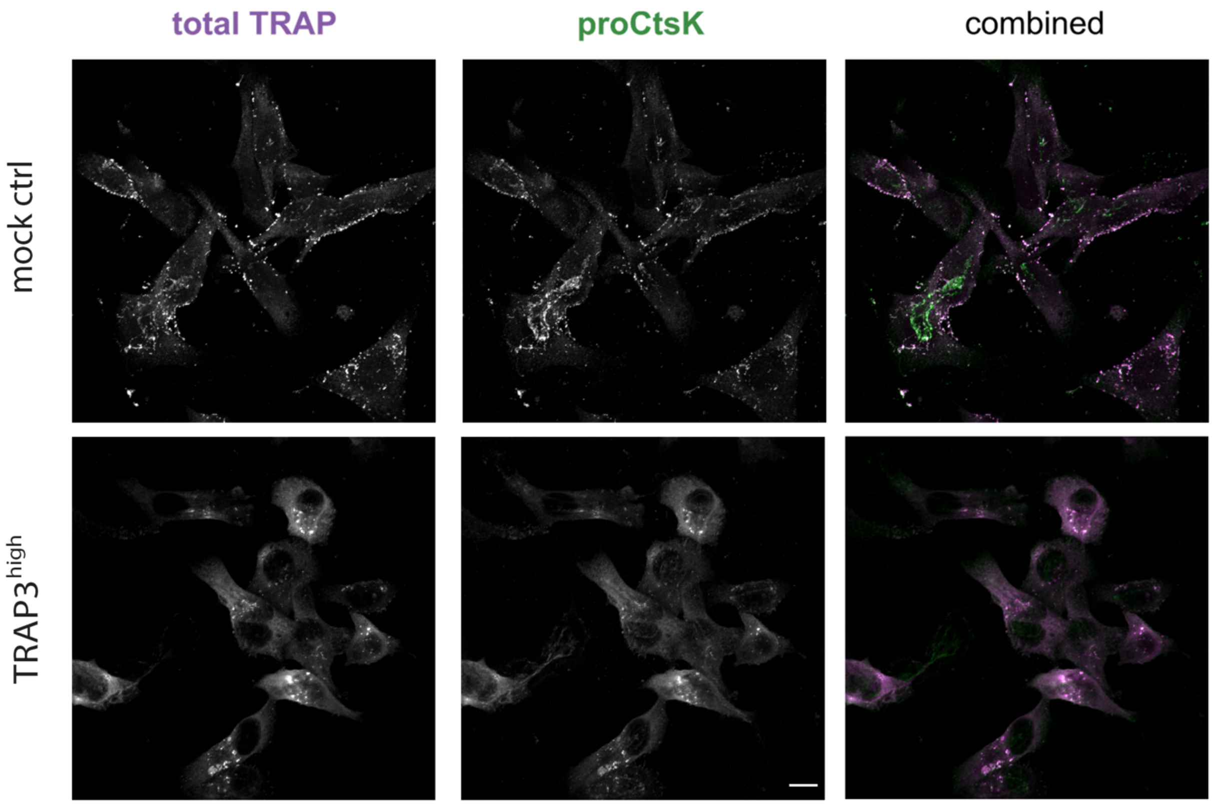

Co-staining of TRAP isoforms and proCtsK in MDA-MB-231 cells treated with MK-0822. ICC staining of proCtsK (Alexa 488, green) and total TRAP (Alexa 647, magenta) of mock control and TRAP3^{high} cells (n=3). Representative single color and merged maximum intensity projections are shown in A and B. Scale bar is 10 µm.

Supplementary Table 1. Fold changes in Western blot quantification of TRAP bands of the different FPLC fractioned cell lysates

**MK-0822 treated vs. DMSO control (fold change)**	F31-33	F36-38	F41-43	F48-50	F53-55
TRAP 5a	2,1	3,3	-	-	-
TRAP 5b N-terminal	1,6	2,6	1,2	2,1	1,2
TRAP 5b C-terminal	1,0	1,3	1,5	1,4	1,2

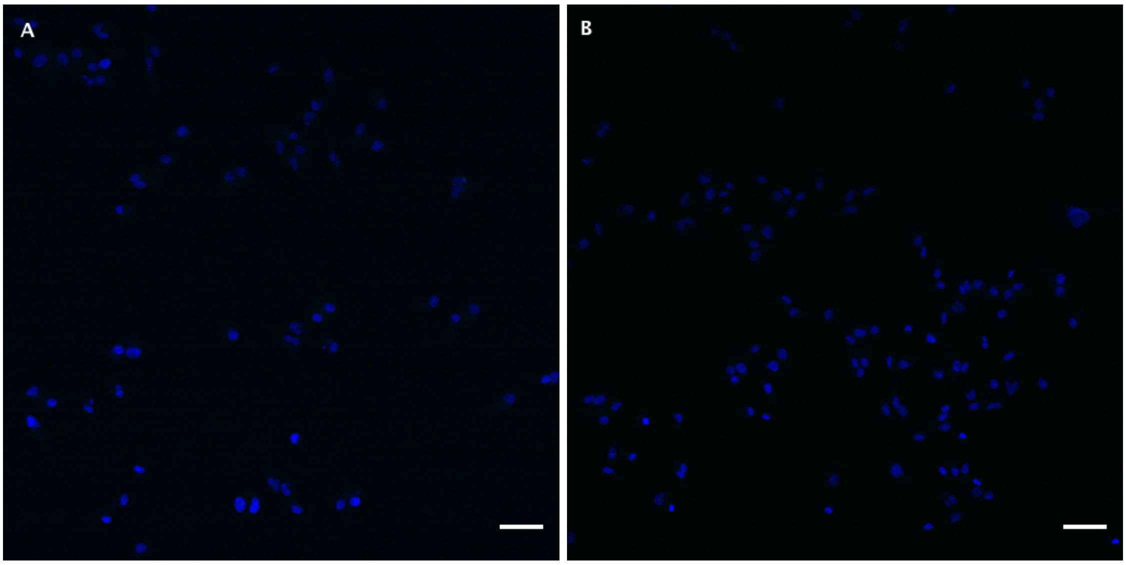

Typical control for nonspecific binding of secondary antibodies. **(a)** mock control and **(b)** TRAP3^{high} cells. Nonspecific staining green, nuclei blue. Scale bar is 50 µm.
